# COVID-19 Vaccination Among Healthcare Workers: Trend and Protection in a Tertiary Care Hospital in Northern India

**DOI:** 10.7759/cureus.35777

**Published:** 2023-03-05

**Authors:** Rashmi Salhotra, Asha Tyagi, Evelyn E Minz, Pragya Chaudhary, Deepak Singh, Venu Toppo

**Affiliations:** 1 Anaesthesiology and Critical Care, University College of Medical Sciences, New Delhi, IND; 2 Anaesthesiology, Vardhman Mahavir Medical College and Safdarjung Hospital, New Delhi, IND; 3 Anaesthesiology, Max Super Speciality Hospital, New Delhi, IND; 4 Preventive Medicine, All India Institute of Medical Sciences, New Delhi, New Delhi, IND

**Keywords:** public health, infection, india, health-care workers, covid-19 vaccines

## Abstract

Background

Coronavirus disease 2019 (COVID-19) is an infectious disease that poses health risks to everyone exposed to the virus and frontline healthcare workers (HCWs) are at very high risk. COVID-19 vaccines have been developed to offer protection from the disease and reduce the severity of illness.

Objective

This questionnaire-based cross-sectional survey aimed to determine COVID-19 vaccination trends and protection among HCWs in a dedicated COVID-19 tertiary care hospital in Northern India.

Methods

A printout of the questionnaire was distributed. Part 1 of the questionnaire included voluntary consent and demographics information, and part 2 dealt with COVID-19 vaccination, COVID-19 illness, and post-vaccination illness. The outcomes of the study comprised trends and protection offered by COVID-19 vaccination, post-vaccination side-effects, and reasons for vaccine hesitancy. The responses were analyzed using Stata version 15.0.

Results:

A total of 256 HCWs were approached to take the questionnaire, out of whom 241 consented to participate in the survey. One-hundred and fifty-five (64.3%) of these HCWs were fully vaccinated, 53 (21.9%) were partially vaccinated, and 33 (13.7%) were non-vaccinated. The overall rate of infection was 45.64% (110/241). The rate of infection was 58.18% among non-vaccinated HCWs, 21.81% after partial vaccination, and 20% after full vaccination. The odds of infection among vaccinated versus non-vaccinated HCWs was 0.338 (95% CI: 0.224 to 0.512; *P*<0.001). The overall hospitalization rate among infected HCWs was 6.36% and there was no incidence of hospitalization among fully vaccinated HCWs.

Conclusions:

Vaccination was shown to reduce the rates of infection and hospitalization among HCWs. A sizeable number of HCWs remained unvaccinated due to either recent COVID-19 infection or apprehension about vaccine-related side-effects.

## Introduction

The coronavirus disease 2019 (COVID-19) pandemic has taken a huge toll since it began in December 2019, claiming many lives worldwide. At that time, vaccination was considered the most appropriate step to address the pandemic. India, being the second most populous country in the world, faced the huge challenge of vaccinating its people. The Government of India rolled out the world’s largest vaccination drive on January 16, 2021, to vaccinate a staggering 300 million individuals belonging to priority groups. The initial beneficiaries were the 10 million healthcare workers (HCWs) directly exposed to severe acute respiratory syndrome coronavirus 2 (SARS-CoV2) [1].

Two vaccines were granted emergency use authorization by the Central Drugs Standard Control Organization (CDSCO) in India, namely, AstraZeneca’s Covishield® (manufactured by Serum Institute of India, Pune, India) and Covaxin® (manufactured by Bharat Biotech International Limited, Hyderabad, India). The overall efficacy of Covishield® was reported to be 70.42% in a preliminary study using data from 23,745 participants aged 18 years or older in overseas clinical studies [2]. Covaxin® demonstrated an efficacy of 77.8% against symptomatic infection, 65.2% against the B.1.617.2 (Delta) variant, and 93.4% against severe COVID-19 illness [3]. As of March 4, 2023, India has administered 220 crore doses of COVID-19 vaccines, which is a notable achievement for any nation [4].

India witnessed a high mortality rate during the second wave of COVID-19 in the months of March-June 2021. The vaccine coverage at that time was a mere 4%, which was most likely due to hesitancy regarding the efficacy and safety of the indigenous vaccines, relative shortages in vaccine supply, and a lack of infrastructure necessary to serve India’s large population [5].

This survey was conducted among HCWs in a dedicated COVID-19 hospital in Northern India to evaluate vaccination against COVID-19 in terms of trends toward acceptance, the protection offered against infection and hospitalization, early post-vaccination side effects, and reasons for vaccine hesitancy.

## Materials and methods

This was a questionnaire-based cross-sectional survey done in August-September 2021. Approval from the Institutional Ethics Committee - Human Research, University College of Medical Sciences, New Delhi, India (approval number: IECHR_2021_50_3) was obtained before starting the survey. HCWs employed by a dedicated COVID-19 hospital, Guru Teg Bahadur Hospital, New Delhi, India, were approached and appraised regarding the survey. Upon voluntary consent to participate in the survey, a printout of the questionnaire was administered, which was available in two languages: Hindi and English. The participant was free to choose the language of the questionnaire.

The questionnaire was divided into two parts: Part 1 included voluntary consent for participation and demographic details such as age, sex, educational qualification, and nature of duties; Part 2 comprised 25 questions with a mixture of open-ended and closed-ended questions. Part 2 was further divided into three sub-parts: (A) COVID-19 vaccination (10 questions), (B) COVID-19 illness (12 questions), and (C) post-vaccination COVID-19 illness (three questions; to be answered only if the HCW had suffered COVID-19 illness after one or two doses of the COVID-19 vaccine), which inquired about the participants’ COVID-19 vaccination status, COVID-19 illness, hospitalization history including the use of oxygen and repurposed drugs, vaccine-related side effects, and barriers to vaccination.

The questionnaire was designed and reviewed by two independent reviewers. A pilot study was conducted with 10 participants each for the English and Hindi questionnaires, and feedback was obtained regarding the clarity of the questions. Necessary changes to the questions and further simplification of language were done as per the feedback to make the questions easily understandable and unambiguous. The responses from participants in the aforementioned pilot study were not included in the main study.

The primary outcome of the main study was determining the trend of COVID-19 vaccination in terms of being fully vaccinated, partially vaccinated, or unvaccinated (i.e., having received two, one, or no doses of the vaccine, respectively), along with the protection offered by the COVID-19 vaccination. Protection was measured in terms of infection and need for hospitalization. Other outcomes included post-vaccination side effects and reasons for vaccine hesitancy.

The responses obtained were compiled in a spreadsheet for statistical analysis using Stata Statistical Software: Release 15 (2017; StataCorp LLC, College Station, Texas, United States). The results are reported as numbers and percentages, and odds ratios were calculated using logistic regression.

## Results

A total of 256 HCWs were approached, out of whom 241 (94%) consented to participate in the survey. The demographic details of the participants are shown in Table 1.

**Table 1:** Demographic profile of the participants (n=241).

Age (years), mean±SD	33.82 ± 9.57
Gender, Male:Female	101:140
Current Educational Status, n (%)	
Uneducated	1 (0.4%)
Schooling	41(17%)
Graduate and diploma	139 (58%)
Post-graduate	60 (25%)
Working Capacity, n (%)	
Doctor	116 (48%)
Technical and support staff	29 (12%)
Nursing staff	83 (34%)
Nursing orderly and cleaning staff	13 (5%)

The majority of participants (n=155, 64.3%) were fully vaccinated, 21.9% were partially vaccinated, and 13.7% were unvaccinated (Table 2). Among the 208/241 HCWs who were fully or partially vaccinated, Covishield® was more commonly received than Covaxin® at 94.7% and 5.3%, respectively.

**Table 2:** Trends of vaccination and the protection offered. HCW: healthcare worker

Vaccination rate (n=241)	
Fully vaccinated, n (%)	155 (64.3%)
Partially vaccinated, n (%)	53 (21.9%)
Unvaccinated, n (%)	33 (13.7%)
Infection, n (%)	
Overall rate of infection, n (%)	110 (45.64%)
Infections in unvaccinated HCWs (n=110), n (%)	64 (58.18%)
Infections in partially vaccinated HCWs, n (%)	24 (21.8%)
Infections in fully vaccinated HCWs, n (%)	22 (20%)
Odds ratio of getting infected after partial vaccination	0.360 (95% CI: 0.216 to 0.602); P <0.001
Odds ratio of getting infected after full vaccination	0.457 (95% CI: 0.268 to 0.780); P =0.004
Hospitalization among infected HCWs (n=110)	
Overall rate of hospitalization, n (%)	7 (6.36%)
Hospitalizations in unvaccinated HCWs, n	4
Hospitalizations in partially vaccinated HCWs, n	3
Hospitalizations in fully vaccinated HCWs, n	0

Details of the protection offered by vaccines are given in Table 2. The overall rate of infection was 45.64% (110/241). Out of these 110 infections, 64 (58.18%) occurred before receiving a first vaccination dose, 24 (21.81%) occurred after partial vaccination, and the remaining 22 (20%) occurred after full vaccination. The odds of infection among vaccinated HCWs was 0.338 (95%CI: 0.2235 to 0.5116; P<0.001). The overall hospitalization rate among infected HCWs was 7/110 (6.36%), with four of these occurring before vaccination, three occurring after partial vaccination, and none occurring after full vaccination.

More than half of the vaccinated HCWs experienced one or more post-vaccination side effects, with fever being the most common, closely followed by bodyache. No serious side effects were noted (Table 3).

**Table 3:** Post-vaccination side effects.

Post-vaccination Side Effects (n=208)	
None	89/208 (42.8%)
One or more side-effects	119/208 (57.2%)
Fever	92/119 (77.3%)
Bodyache	80/119 (67.2%)
Weakness	45/119 (37.8%)
Injection site pain	6/119 (5.0%)
Diarrhea	3/119 (2.5%)

**Figure 1:**
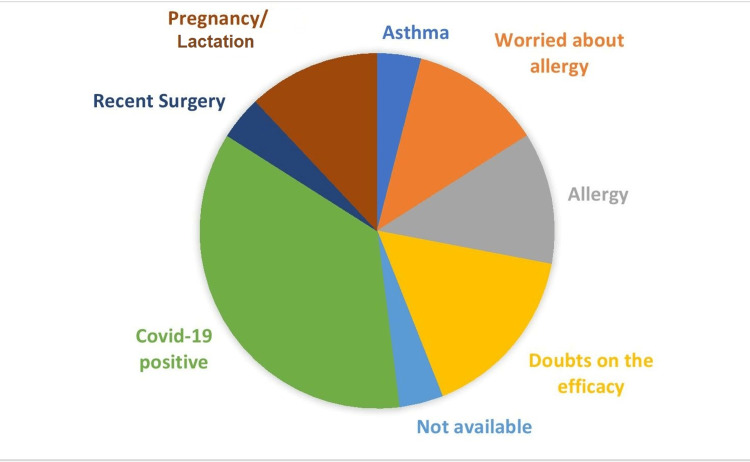
Reasons for vaccine hesitancy (n=33, 13.69%).

A total of 33 out of 241 HCWs (13.7%) had not been vaccinated with a COVID-19 vaccine. The major reason for vaccine hesitancy in these participants was a recent COVID-19 illness (36%), i.e. within four weeks, followed by doubts regarding vaccine efficacy (16%), allergic predisposition (12%), or being worried about the possibility of allergic reaction to the vaccine (12%) (Figure 1).

## Discussion

Vaccination against COVID-19 has not been accepted universally with full enthusiasm. Vaccine hesitancy and refusal have been causes of concern among the population at large, including frontline HCWs. One study in China reported that 18.28% of HCWs were hesitant and 4.74% were resistant to the idea of receiving vaccination [6]. In the United States, vaccination coverage among HCWs was reported to be 70% by mid-September 2021 [7], whereas an astounding 66.73% of HCWs had not even enrolled themselves for vaccination in the Kingdom of Saudi Arabia in the first month that the vaccine was available [8]. At the time of initiation of this survey, there was little data on vaccination among the Indian HCWs. Our study was conducted during the second wave of the pandemic in the months of August-September 2021 when the vaccination drive in India was in its ninth month. This survey was conducted among HCWs of a tertiary care teaching institute that had been converted to a dedicated COVID-19 care facility and was catering to 1500 patients. We found that 64.3% of HCWs were fully vaccinated, and another 21.9% were partially vaccinated; unvaccinated HCWs constituted 13.7% of the workforce. The institution where the survey was done was a vaccination site under the government’s vaccination program, and Covishield® was the only vaccine being offered at this facility, thus explaining why 94.7% of the HCWs had received this vaccine.

Right from the beginning of the vaccination drive, it had been clarified that the efficacy of the vaccine is not 100%. However, vaccination has been shown to reduce the chances of infection and the severity of disease, and, consequently, the need for hospitalization [9]. One study in Italy reported that the risk of COVID-19 infection in unvaccinated HCWs was 17.8%, and the risk of reinfection was 3%; the rate of infection decreased to 1.5% among vaccinated HCWs, with the duration of infection being as short as two days and the majority of cases being asymptomatic or developing mild disease [10]. The results obtained in our survey also showed that the overall rate of infection was 45.6%. Out of these cases, 58.18% of infections were seen in unvaccinated HCWs. However, after partial vaccination, the rate of infection went down to 21.8% of total infections, and then to 20% after full vaccination. The odds of infection after vaccination was significantly lower than before vaccination (P<0.001).

Vaccination was associated with reduced odds of hospitalization compared to that in unvaccinated individuals in a prospective community-based, nested, case-control study [11]. In our study, the overall hospitalization rate among infected HCWs was 6.36%, and there was no incidence of hospitalization among HCWs who were fully vaccinated.

In our survey, 42.78% of participants did not have any post-vaccination side effects. In the 57.21% HCWs who developed one or more post-vaccination side effects, fever and bodyache were the most common, followed by weakness, injection-site pain, and diarrhea. Ninety-seven out of the 119 HCWs (81.5%) who reported side effects experienced them after their first vaccination dose, and only 15.13% reported side effects after both vaccine doses. No serious side effects were noted in this small-sample survey. Fever, chills, bodyache, and other systemic manifestations after vaccination are common side effects of other viral vaccines as well [12,13], which are due to the activation of the body’s immune response to the foreign pathogen [14].

As per a nationwide survey conducted among the general population in India in December 2020, 37% of individuals were not interested or were not sure whether they wanted to receive the vaccine. The major concerns with the vaccine were related to its efficacy and safety, as well as the requirement of vaccination if an individual had already been infected with COVID-19 [15]. An online survey conducted in the beginning of 2022 revealed that among the unvaccinated citizens of India, 27% (i.e., around 7% of the country’s population) were hesitant in taking the COVID-19 vaccines [16]. Our study found that 13.69% of HCWs were yet to receive the vaccine, with the major reason being that a majority of them (36%) had suffered from a recent COVID-19 infection and were waiting for a three-month time gap between the infection and the vaccine shots. These HCWs had planned to receive the vaccine. Of those unwilling to be vaccinated, 16% were hesitant due to doubt regarding the efficacy of the vaccine, and worry about allergic manifestations and history of allergy each constituted 12%.

The main limitations of the study include the fact that this was a single-centre study and it was conducted in the early months of the vaccine being released so hesitancy would be more. Larger multi-centre studies should be done at a later date so that a better and more recent picture is got for comparison.

## Conclusions

Based on the findings of our survey, COVID-19 vaccines were found to be effective in reducing the incidence of COVID-19 infection. COVID-19 infection in vaccinated individuals is mild and rarely requires hospitalization. A majority of HCWs in our study had taken the vaccination by September 2021. However, 13.7% of HCWs still remained unvaccinated, out of which a sizeable number could not be vaccinated due to a recent history of COVID-19 infection. The major barrier to vaccination was doubt regarding its efficacy. We hope that as such hesitancy decreases among the general population, these HCWs would also be eventually convinced about the vaccine’s efficacy.
